# 复合性小细胞肺癌伴*EGFR*基因突变1例报道及治疗体会

**DOI:** 10.3779/j.issn.1009-3419.2014.06.14

**Published:** 2014-06-20

**Authors:** 晔 郭, 丽梅 曲, 铭心 邵, 星星 王, 宏伟 孙, 克威 马

**Affiliations:** 1 130021 长春，吉林大学第一医院肿瘤中心 Caner Center, the First Hospital of Jilin University, Changchun 130021, China; 2 130021 长春，吉林大学第一医院病理科 Department of Pathology, the First Hospital of Jilin University, Changchun 130021, China

复合性小细胞肺癌（combined small cell lung cancer, CSCLC）是小细胞肺癌（small cell lung cancer, SCLC）与另外一种成分复合组成的癌，世界卫生组织2004年肺癌新病理分类中，将其归为SCLC的亚型。CSCLC发生率低，约占SCLC的1%-2%^[1]^，目前尚无标准治疗方案，疗效和预后有待进一步观察。本文报道1例CSCLC，复合成分为腺癌，且经过ARMS法检测肿瘤组织表皮生长因子受体（epidermal growth factor receptor, *EGFR*）突变阳性，该患者经过化疗、分子靶向治疗及维持治疗，取得良好的治疗效果。

## 1 临床资料

患者男性，61岁，吸烟30余年，40支/日。咳嗽、咳痰1个月。既往：患者5年前体检发现胸膜处高密度阴影（图 1A），5年间无明显变化，发病前3个月病灶逐渐增大。辅助检查：2013年2月肺CT（图 1B）：右肺下叶见团块状密度增高影，呈浅分叶并见偏心性空洞，大小约51 mm×47 mm；右侧胸膜见多个结节状密度增高影，右侧胸腔内见少量液性低密度影；纵隔内气管隆突上、下及右侧心隔角见多个肿大淋巴结，部分融合成块。行右肺下叶病灶穿刺，病理回报：肺复合性小细胞癌，复合成份为腺癌。镜下观察，肿瘤组织由两类细胞组成，其中以弥漫蓝染的小细胞分布为主，细胞排列紧密，大部分呈短梭形，包浆稀少，易见核分裂像，其内穿插少量紊乱腺腔样排列的肿瘤细胞，细胞有异形。免疫组化：CK7（+），Syn（+），TTF-1（+），CD56（+），CK5/6（-），CK20（-），P63（-），CgA（-），LCA（-），Ki-67（50%+）（图 2）。穿刺组织行*EGFR*基因突变检测：第19号外显子有缺失突变。

**1:**
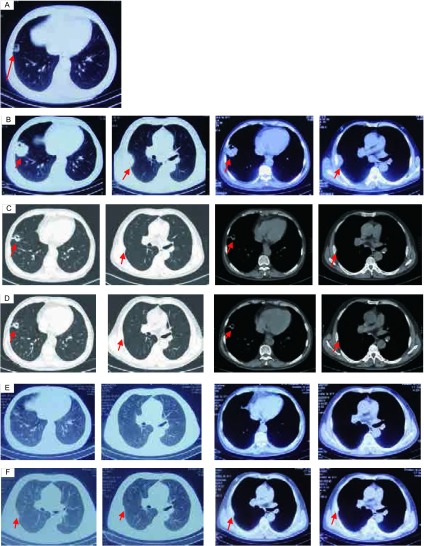
CT图像。A：患者发病5年前体检时肺CT；B：患者发病初始肺CT（2013年2月）；C：2个周期化疗后肺CT（2013年4月）；D：4个疗程化疗后肺CT（2013年5月）；E：6个疗程化疗+分子靶向治疗后肺CT（2013年7月）；F：分子靶向药物维持治疗2个月后肺CT（2013年9月）。 CT graphs. A: patient's pulmonary CT before 5 years; B: Patient's pulmonary CT at the beginning of the disease (February, 2013); C: patient's pulmonary CT after 2 cycles of chemotherapy (April, 2013); D Patient's pulmonary CT after 4 cycles of chemotherapy (May, 2013); E: Patient's pulmonary CT after 6 cycles of chemotherapy and TKI treatment (July, 2013); F: Patient's pulmonary CT by TKI as maintenance treatment after 2 months (September, 2013). CT: computed tomograpy; TKI: tyrosine kinase inhibitor.

**2:**
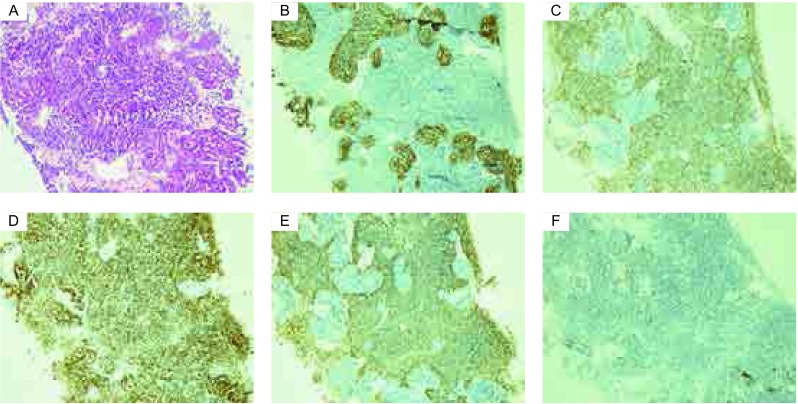
患者肿瘤组织的HE和免疫组化染色结果（×200）。A：HE染色；B：腺癌成分CK7表达阳性，细胞膜表达；C：小细胞癌成分Syn表达阳性，细胞膜表达；D：腺癌、小细胞癌成分TTF-1均表达阳性，细胞核表达；E：小细胞癌成分CD56表达阳性，细胞膜表达；F：CK5/6表达阴性。 HE and immunohistochemical staining of the patient's tumor tissue (×200). A: HE staining; B: Positive material of CK7 is located on the adenocarcinoma cell's membrane; C: Positive material of Syn is located on the small cell carcinoma cell's membrane; D: Positive material of TTF-1 is located on the adenocarcinoma cell's and small cell carcinoma cell's nuclei; E: Positive material of CD56 is located on the small cell carcinoma cell's membrane; F: Expression of CD5/6 is negative.

患者已存在胸膜转移，肿瘤分期较晚，无手术机会，治疗策略采用姑息治疗为主。一线治疗选择：伊立替康联合顺铂，即IP方案。2个周期化疗后复查肺CT疗效评价为部分缓解（partial response, PR）（图 1C）。继续给予原方案化疗，第4周期化疗后复查肺CT：疗效评价仍为PR。但对比2个疗程化疗后靶病灶未见明显变化（图 1D）。在原有化疗方案基础上加用分子靶向治疗，即小分子酪氨酸激酶抑制剂（tyrosine kinase inhibitor, TKI），方案为IP+盐酸埃克替尼（125 mg/次，3次/日，d1-21口服），21天为1个周期。2个周期该方案化疗后疗效评价为完全缓解（complete response, CR）（图 1E）。继续给予患者盐酸埃克替尼（125 mg/次，3次/日）口服进行维持治疗。维持治疗两个月后，患者胸膜结节影缓慢进展（图 1F），无明显临床症状。二线治疗方案选用拓扑替康+盐酸埃克替尼，21天为1个周期。4个周期治疗后疗效评价为稳定（stable disease, SD）。停止化疗，继续给予盐酸埃克替尼维持治疗，疾病控制稳定。自患者发病至今已10个月。

## 2 讨论

两种或两种以上类型的癌组织成分混合构成的癌，统称为复合性癌。复合性癌的发病率约占恶性肿瘤的0.3%-0.5%^[2]^。CSCLC是SCLC与另外1种成分复合组成的癌。这种复合成分可以是任何类型的非小细胞肺癌（non-small cell lung cancer, NSCLC），通常为腺癌、鳞癌或大细胞癌，少数为梭形细胞或巨细胞癌，甚至含有肉瘤样成分。SCLC在肺癌中恶性程度最高，约占肺癌的15%-20%，肺腺癌约占肺癌的20%-25%。在亚洲人群中肺腺癌*EGFR*突变率约为30%-45%^[3]^。因此本文报道的CSCLC伴*EGFR*基因突变病例极为少见。

针对CSCLC的治疗，主张以化疗为主的综合治疗，并无统一规范治疗方案。本病例一线治疗的选择主要考虑以下几点：①SCLC对化疗敏感，疾病控制率可达85%-95%。肺腺癌化疗客观缓解率约为35%-45%，*EGFR*基因突变阳性腺癌，应用TKI药物的客观缓解率可高达70%^[4]^；②*EGFR*突变常见于肺腺癌细胞内，罕见于SCLC，若选择TKI类药物可能对SCLC治疗无效；③本病例病理证实以SCLC成分为主，且SCLC倍增时间短、恶性程度高、具有高度侵袭性，故治疗上应选针对SCLC效果确切且对于NSCLC亦有效的化疗方案^[5]^。2个周期化疗后肺部病灶明显缩小，疗效评价为PR，患者咳嗽、咳痰症状消失，可见该化疗方案的选择可使患者获益。4个周期化疗后复查肺CT对比前片未见明显变化。考虑SCLC成分已有效控制，继续治疗应选用针对腺癌成分的方案，同时考虑肿瘤组织*EGFR*突变阳性，应用TKI药物的客观缓解率高于化疗^[4]^，故在化疗基础上加用TKI分子靶向治疗，治疗2个周期后患者达CR。由此可见，针对该类复合性肿瘤，化疗联合分子靶向治疗可取得理想效果。该患者6个周期化疗结束后，继续给予分子靶向药物维持治疗。

患者复发后选择二线治疗方案时考虑：主要为小细胞成分复发，但不能排除存在腺癌成分，故给予SCLC二线化疗方案基础上联合分子靶向治疗，4个周期化疗后疾病控制稳定，继续给予分子靶向药物维持治疗。目前该患者疾病控制稳定，关于后续治疗方案的选择及疗效，有待继续跟踪报道。
